# Molecular mechanisms of tamoxifen-associated endometrial cancer (Review)

**DOI:** 10.3892/ol.2015.2962

**Published:** 2015-02-12

**Authors:** RONG HU, LEENA HILAKIVI-CLARKE, ROBERT CLARKE

**Affiliations:** Department of Oncology, Lombardi Comprehensive Cancer Center, Georgetown University, Washington D.C. 20057, USA

**Keywords:** Tamoxifen, endometrial cancer, estrogen receptor, G protein-coupled receptor 30, unfolded protein response, autophagy

## Abstract

Tamoxifen has been prescribed to millions of females for breast cancer prevention or treatment. However, tamoxifen is known to significantly enhance the risk of developing endometrial lesions, including hyperplasia, polyps, carcinomas, and sarcoma. Notably, tamoxifen-associated endometrial cancer often has a poor clinical outcome. Understanding the molecular mechanism of tamoxifen-induced endometrial cancer is essential for developing strategies that minimize tamoxifen’s effects on the endometrium without jeopardizing its breast cancer treatment effects. However, this understanding remains limited. Tamoxifen appears to mediate its effect on endometrial cells through estrogenic and non-genomic pathways, rather than introducing a genomic alteration as a carcinogen. Although tamoxifen functions as an agonist and promotes cell proliferation in endometrial cancer, it also displays antagonist activity towards some estrogen targets. Alterations in estrogen receptor-α and its isoforms, as well as the membrane associated estrogen receptor G protein-coupled receptor 30, have been observed with tamoxifen-exposed endometrial cells, and likely mediate the effects of tamoxifen on endometrial cancer cell proliferation and invasion. In addition, gene profile studies of short-term exposure to tamoxifen indicate that the majority of tamoxifen targets are tamoxifen-specific. However, the tamoxifen regulated gene targets that are involved in mediating the effects of long-term exposure to tamoxifen are not yet fully understood. Recent progress has indicated a potential role of unfolded protein response and mammalian target of rapamycin signaling in tamoxifen-associated endometrial cancer. In the future, studies focusing on long-term effects of tamoxifen exposure are required to understand the molecular mechanisms of tamoxifen-associated endometrial cancer.

## 1. Introduction

Tamoxifen remains a first-line adjuvant treatment for premenopausal breast cancer patients with estrogen receptor-α (ERα) positive tumors, and is often prescribed to postmenopausal females with ERα+ tumors. Tamoxifen functions as an antagonist to ERα and blocks its signaling pathway in ERα+ breast cancer cells. The risk of recurrence of breast cancer and the risk of contralateral breast cancer are significantly reduced by tamoxifen treatment. Five years of tamoxifen treatment reduced the risk of relapse after 10 years by 37% in females aged 50–59 years, and by 54% in females aged 60–69 years. Recently, a study showed that for patients with ERα+ breast cancer, continuing tamoxifen to 10 years rather than discontinuing at five years produces a further reduction in recurrence and mortality (1). Following its introduction in the 1970s, tamoxifen has been prescribed to millions of patients with ERα+ breast cancer, which accounts for ~70% of all breast cancer patients (2). Tamoxifen is now available as a generic drug worldwide, which has made it the single most prescribed drug in the world for the treatment of any cancer (2). In 1998, the USA Food and Drug Administration further approved tamoxifen as a breast cancer prevention drug for high-risk patient groups, increasing the number of females that are prescribed with tamoxifen even further.

Although tamoxifen is an extremely effective treatment for breast cancer, this drug also has serious side effects. One of the most significant and detrimental side effects of postmenopausal tamoxifen treatment is its ability to increase a patient’s chance of developing endometrial lesions, including hyperplasia, polyps, carcinomas, and sarcoma (3). The increased risk of endometrial cancer varies between studies, ranging from 1.5 to 6.9-fold (4). The risk of endometrial cancer is not associated with the daily dosage of tamoxifen, but with longer duration and accumulative usage (5). The risk of endometrial cancer in tamoxifen users increases significantly with increasing body weight among postmenopausal females (6).

Independent from tamoxifen intake, breast cancer patients have an enhanced risk of endometrial proliferative disorders, including hyperplasia, as indicated by the high prevalence of this pathology detected in breast cancer patients undergoing endometrial assessment prior to the initiation of tamoxifen administration (7,8). In a recent study, the baseline hysteroscopic assessment revealed an incidence of 31.3% of overall endometrial morbidity in asymptomatic postmenopausal ERα+ breast cancer patients (9). For patients who have endometrial conditions such as hyperplasia without atypia prior to tamoxifen treatment, the rate of progression to more advanced stage endometrial lesions is almost 50% after 24 months of tamoxifen administration (10).

Initially, endometrial cancers induced by tamoxifen exposure were considered tumors with good prognosis. However, more recent studies have found these endometrial cancers to have a relatively poor prognosis. Endometrial cancer in tamoxifen users often belongs to the less favorable morphological subtypes, and thus may have an increased mortality (1). The three-year endometrial cancer specific survival significantly decreases from 94% for non-users to 76% for tamoxifen users of more than five years (5). In a large case-control study on the risk and prognosis of endometrial cancer following tamoxifen use for breast cancer, endometrial cancers of stage III and IV occurred more frequently in long-term tamoxifen users than non-users. In addition, long-term users experience significantly higher risks of developing malignant mixed mesodermal tumors or sarcomas of the endometrium than those not taking tamoxifen (15.4% vs. 2.9%; P<0.02). Breast cancer survivors whose endometrial carcinoma was of a high risk histological type had a longer median duration of prior tamoxifen use compared with that of those with lower risk histological types (11).

As the net benefit of tamoxifen greatly outweighs the risk, the worldwide usage of tamoxifen for breast cancer patients is expected to continue, particularly as a generic drug. Therefore, lowering the risk of endometrial cancer for tamoxifen users is an increasingly important cancer prevention target. Unfortunately, since the adverse effects of endometrial cancer in tamoxifen users were reported in 1997, the current understanding of how exposure to tamoxifen affects endometrial tissue and induces endometrial cancer remains limited, despite several mechanisms being proposed. This review summarizes the current view of the possible mechanisms involved and provides an outlook for the future studies towards the prevention of development of endometrial cancer in tamoxifen users.

## 2. Molecular mechanisms for tamoxifen-induced endometrial cancer

Unlike the majority of ERα+ breast cancer cells, tamoxifen induces cellular growth in endometrial cancer cells *in vitro*. Several signaling pathways that promote cell proliferation, including mitogen-activated protein kinase (MAPK) pathways, c-MYC and insulin-like growth factor 1 (IGF1) pathways, were elevated upon tamoxifen exposure (12). Consistent with the effects observed in *in vitro* cell culture, tamoxifen exposure promotes endometrial cell proliferation *in vivo*. A single injection of tamoxifen strongly induced an increase in uterine wet weight and proliferation in the endometrium at 16 h post-injection in mice (13). A robust induction of proliferation markers, including pRb, cyclin D, cyclin E, cyclin A and cyclin-dependent kinase (CDK) 2 was observed at 8–16 h post-injection; a decrease to basal level occurs by 48 h (14). Clinical data comparing the endometrium of tamoxifen users and non-users also indicated that exposure to tamoxifen promotes endometrial proliferation. The apoptosis/proliferation index, determined by measuring the proliferation marker Ki67 and the apoptosis/anti-apoptosis markers Fas, FasL and Bcl2, is higher in the benign endometrial lesions of tamoxifen users compared with those of non-users (15,16). Recently, the BH3 mimetic drug ABT-737 was observed to partially counteract tamoxifen-induced endometrial hyperplasia (17). The combined administration of ABT-737 with tamoxifen to severe combined immunodeficiency mice for 10 days reversed the increase in uterine weight induced by tamoxifen treatment only, possibly via the promotion of apoptosis. However, the molecular mechanism of the effects of BH3 mimetics in blocking tamoxifen’s effects requires further investigation.

In addition to cell proliferation, tamoxifen has been shown to promote cytoskeletal remodeling and migration in endometrial cancer cells. Tamoxifen exposure induces focal adhesion kinase (FAK) phosphorylation via extracellular-signal-regulated kinases (ERK) and Src signaling, and thus promotes migration (18). The effects of tamoxifen on cell migration appear to be ER signaling dependent. In ERα positive Ishikawa cells, inhibition of ERα blocks the migration effects of tamoxifen, whereas in ER negative RL95-2 endometrial cancer cells, migration is mediated through G protein-coupled receptor 30 (GPR30) and ERK/FAK pathway (19). Notably, tamoxifen not only promotes invasion of endometrial cancer cells, it more than doubles the invasion of endometrial stromal cells in a three-dimensional coculture model (20). Paracrine factors released from endometrial stromal cells are able to promote epithelial proliferation of endometrial cells (21), emphasizing the importance of stromal cells in endometrial carcinogenesis. These results clearly demonstrate the role of tamoxifen on endometrial stromal cells, and raise the possibility that tamoxifen promotes endometrial cancer partially through its effects in the stroma.

## 3. DNA damage induced by tamoxifen

Tamoxifen is metabolized to an array of metabolites with estrogenic effects, and also to reactive intermediates that may form protein or DNA adducts to cause DNA damage. Therefore, it has been hypothesized that tamoxifen induces malignancies by its genotoxicity. Tamoxifen is known as a strong liver carcinogen in rats (22–25), and a high frequency of p53 mutations is detected in hepatocarcinomas induced by tamoxifen exposure (26). Intraperitoneal administration of tamoxifen to female mice leads to the formation of hepatic DNA adducts (27). However, analysis of tamoxifen (Tam)-DNA adducts in endometrial tissues from patients treated with tamoxifen has yielded mixed results. Several studies failed to detect Tam-DNA adducts in endometrial tissue (28,29), in contrast with a number of other studies (30,31). In addition, Tam-DNA adduct formation has been detected in glandular and surface epithelia following the incubation of human endometrial explants with tamoxifen (32). While Tam-DNA adduct formation is possible in human tissues, compelling evidence that this drives endometrial cancer is lacking. Furthermore, the risk of developing hepatocellular cancer is minimal in females treated with tamoxifen, and Tam-DNA adduct formation in endometrial tissue occurs at an extremely low level and in only a few females. Consistent with the lack of DNA damage effects on tamoxifen-associated endometrial tissue, a large scale chromosomal comparative genomic hybridization analysis comparing the genomic profile of endometrial tumors from long-term (>2 year) tamoxifen exposed and unexposed breast cancer patients revealed that the genomic aberrations are indistinguishable in the two groups (33). Instead, genomic profiles are correlated with morphological subtypes of the endometrial tumors (33). Therefore, the importance of genotoxity as a major pathway for tamoxifen-induced endometrial cancer is unclear.

## 4. Effects of tamoxifen on driver genes of sporadic endometrial cancer

Several genes have been shown to be associated with sporadic endometrial cancer. One of the most common genetic alterations is the mutation or loss of heterozygosity in the phosphatase and tensin homolog (PTEN) gene, which occurs in 35–50% of type I endometrial carcinoma. Other common DNA alterations include increased microsatellite instability (MSI), due to defects in DNA mismatch repair family genes, and gene mutations in K-ras (34), β-catenin and p53. Mutation of the K-ras protooncogene occurs most frequently in codons 12 and 13 of exon 1, and has been detected in 4.5–23% of endometrial hyperplasia and in 18–26% of endometrial carcinomas (35,36). β-catenin mutations are present in 14–44% of type I endometrial cancers, and mutations in MSI are identified in 20–40% of type I endometrial carcinomas, and frequently coexist with PTEN mutations. Sequencing and analysis of genetic mutations of these genes in tissue samples of tamoxifen-associated and sporadic endometrial cancer patients have been conducted by a number of research groups. In the majority of cases, patients with tamoxifen exposure had similar mutation rates in PTEN, p53, and MSI genes as non-exposed females with endometrial cancer (37–40).

The frequency of K-ras mutation in tamoxifen-related endometrial polyps is controversial; the incidence was reported to be high (43–64%) in certain cases compared with the 4.5–23% observed with non-users (41,42). Furthermore, the presence of the K-ras mutation is significantly affected by the duration of tamoxifen treatment (43). Conversely, in other reports, no significant difference was observed between tamoxifen*-*treated and non-treated patients (37–39). Similarly, higher p53 mutation or overexpression rates were reported in several studies (5,39), but not in others (44). β-catenin mutation rates may be elevated in tamoxifen-treated females (38), however, the results are also inconsistent. The likelihood of tamoxifen increasing endometrial cancer rate by enhancing mutations of driver genes for sporadic endometrial cancer is low. Instead, long-term tamoxifen exposure is likely to promote endometrial carcinogenesis predominantly via non-genomic alterations. It is possible that tamoxifen offers endometrial cells that contain pre-existing mutations a growth advantage via estrogenic and epigenetic alterations. The result of these activities is a significant increase in the incidence of endometrial cancer in long-term tamoxifen users.

## 5. Estrogenic effects of tamoxifen

In breast cancer cells, tamoxifen acts as an ER antagonist by competing with estrodiol for binding, and by inducing conformational changes that block the interaction of ER with co-activator proteins (45). However, as a selective estrogen receptor modulator (SERM), tamoxifen behaves as an antagonist and/or agonist of ER, depending on the target tissue, and can modulate the signal transduction pathways of estrogen-responsive genes. In endometrial tissue, tamoxifen is known to exert estrogenic actions.

One mechanism that may explain the antagonistic and agonistic effects of tamoxifen in different target tissues is the differential recruitment of co-regulators to the ER target gene promoter. In breast cancer cells, tamoxifen induces the recruitment of co-repressors nuclear receptor co-repressor and silencing mediator for retinoid and thyroid hormone receptors to ER target promoters (12). However, in the endometrium, tamoxifen recruits the co-activators steroid receptor co-activator-1 (SRC-1), amplified in breast cancer-1 (AIB1) and CREB-binding protein (CBP), rather than co-repressors, to ER target gene (12). Inhibition of SRC-1 in Ishikawa cells eliminated tamoxifen-induced gene expression (12). This differential co-regulator recruitment appears to be limited to ER targets that do not contain a classical estrogen response element (ERE) in their promoters, including c-MYC and IGF1 (12). The tissue-dependent mode of action of tamoxifen may be explained by the relative abundance of co-factors in different tissues (46). The expression level of co-regulators such as SRC-1 is low in MCF7 breast cancer cells compared with endometrial Ishikawa cells (12). In addition, SRC-1 activity is regulated by Src kinase, which is highly activated in endometrial cancer cells compared with breast cancer cells (47). However, this mechanism only partially explains the differences observed between breast and endometrial cancer cells, as tamoxifen did not display agonistic effects in all estrogen target genes, indicating the involvement of other molecular mechanisms.

As well as affecting the transcriptional activity of ERα, tamoxifen has been shown to regulate expression levels of ERα in the endometrium. Several studies have demonstrated that the expression of ERα in benign endometrium is higher in breast cancer patients who use tamoxifen compared to non-tamoxifen-users (48,15), thereby promoting estrogen-mediated cell growth. Conversely, decreased ERα expression on endometrial cancer has also been reported. Over 60% of long term tamoxifen users are ERα negative in endometrium, whereas only 26.2% of non-users are ERα negative (5). This observation is consistent with the evidence that mice orally dosed with tamoxifen during early development showed a marked decrease in expression of ERα, with no or very weak staining in the endometrium, stroma and myometrium (49). An explanation for this discrepancy may be that, in the cells with downregulated ERα, upregulation of other ER splice variants or isoforms by tamoxifen exposure compensated for the loss of ERα. ER-α36, a variant form of ERα that is localized to the plasma membrane, has been shown to be upregulated in endometrial cancer cells (50). The expression of ER-α36 increases with tamoxifen exposure in endometrial cancer cells (50). In addition, ER-α36 mediates the tamoxifen-induced MAPK and Akt pathways, and is essential for tamoxifen-stimulated endometrial cell growth (50).

Other than activating nuclear estrogen receptors to induce downstream genomic signaling, estrogen is also known to activate rapid non-genomic signaling events independently of nuclear ER. GPR30, an orphan G-protein-coupled receptor, has been proposed to be a new membrane-bound estrogen receptor involved in the rapid non-genomic effects of estrogen. GPR30 activation leads to the release of heparin-bound growth factor (HB-EGF), which in turn triggers the human epidermal growth factor receptor (EGFR)/MAPK transduction pathway (51). In endometrial cancer, overexpression of GPR30 occurs more frequently in high-grade and advanced stage tumors, and is correlated with poor prognosis (52).

Tamoxifen acts as an agonist for GPR30 to stimulate cell proliferation and growth. GPR30 mediates tamoxifen’s stimulatory effects on endometrial carcinoma cells *in vitro*, possibly through stimulation of MAPK phosphorylation (53). Inhibition of GPR30 eliminates the tamoxifen-stimulated proliferation of endometrial cancer cells (53). More importantly, a significant correlation between GPR30 expression and tamoxifen-induced endometrial pathology has been identified (54). A cohort study comparing the expression pattern of GPR30 in endometrial tissue from breast cancer patients who received tamoxifen, or another adjuvant therapy, showed that the intensity of GPR30 expression was significantly correlated with the time between the initiation of tamoxifen treatment and the development of an endometrial abnormality. Among tamoxifen-treated breast cancer patients, 43.8% exhibited high-grade GPR30 staining, whereas only 13.6% were observed in control group (54).

## 6. Tamoxifen-regulated genes exhibit an overlapping but distinct profile compared with the target genes of estradiol

Studies utilizing different model systems have been conducted to systematically determine the molecular pathways affected by tamoxifen and estradiol *in vitro* and *in vivo*. These studies have indicated that, although there is an overlap between the tamoxifen- and estradiol-induced gene expression profiles, tamoxifen also regulates its own specific set of genes in endometrial cells.

In a study by Tamm-Rosenstein *et al* (55), the endometrial Ishikawa cancer cell line was treated with tamoxifen or estradiol for 12 h, followed by RNA-sequencing. Tamoxifen exposure altered the expression of 1013 genes. Among them, only 224 (22%) genes overlapped with estradiol treatment; all others were tamoxifen-specific gene targets, in pathways including DNA replication, recombination and repair, in addition to cell cycle progression, and cellular assembly and organization. Notably, while tamoxifen is often considered an agonist to estrogen in endometrial cells, the transcriptome results indicate that tamoxifen is both antagonistic and agonistic in the Ishikawa cell line. Similarly, a study utilizing primary cultures of human endometrial epithelial cultures showed that the majority of genes altered by tamoxifen treatment for 24 hrs were estrogen-independent (56). Fong *et al* (13) injected a single dose of tamoxifen or ethinylestradiol to immature ovariectomized mice, and observed a significant increase in uterine wet weight after 24 h. Microarray analysis revealed that tamoxifen and ethinylestradiol target genes overlapped but tamoxifen also independently regulated genes associated with cell growth and proliferation, cytoskeletal organization, extracellular matrix modification, nucleotide synthesis, DNA replication, protein synthesis and turnover, lipid metabolism, glycolysis and immunological responses (13). Furthermore, microarray analysis of endometrial patient tissues revealed that 67% of tamoxifen-regulated genes overlap with estradiol treatment (13). Notably, these investigators found that tamoxifen uses the same set of cell cycle genes as estradiol to promote cell proliferation in endometrial tissue, however, it does so to a lesser extent (57).

The majority of experiments aimed at understanding the mechanism of tamoxifen-induced endometrial cancer are performed with short exposure to tamoxifen, ranging from 16–72 h post single administration. The results obtained through these methods aid in the identification of immediate targets of tamoxifen on endometrial cells. However, the association of endometrial cancer incidence rate with duration of tamoxifen exposure indicates that long-term exposure of tamoxifen has additional promoting effects on endometrial carcinogenesis. As endometrial carcinogenesis is a progressive and not a sudden event, short-term exposure of endometrial cells to tamoxifen is not likely to reveal all of the signaling pathways that are critical for tamoxifen-promoted endometrial cancer. To understand the effects of long-term tamoxifen exposure on endometrial tissue, analyses of gene expression profile from large scale clinical data and experiments with long-term treatment of tamoxifen are required.

To date, only small scale studies comparing the gene expression profiles of tamoxifen-associated and sporadic endometrial cancers from patient samples have been undertaken. In one study, no differences were observed in the gene-expression profile when tamoxifen-induced cancers were matched for grade and stage to non-tamoxifen-associated endometrial cancers; this may have been due to the small number of tumor samples (58). However, another study with stage-matched patient tissue microarray revealed that gene expression profiles in endometrial tumors are different in tamoxifen users compared with non-users (59). Unsupervised clustering of all genes in all samples revealed that tumors from patients who had used tamoxifen clustered together, and were separate from samples of those who had never used tamoxifen. This study identified 256 differently expressed genes, the majority of which were tamoxifen-specific. Notably, a number of these gene products, including p53, RelA, MYC, EGFR and β-catenin, are critical components of pathways that, based on their normal physiological functions, may be important in endometrial carcinogenesis (59). Furthermore, these genes have also been shown to be upregulated and modulate tamoxifen-resistant breast cancer cells, indicating that long-term treatment with tamoxifen may stimulate similar gene networks to promote cancer progression in endometrial cells and overcome sensitivity in breast cancer cells.

## 7. UPR-mTOR-autophagy signaling pathway

The study of endocrine resistance in breast cancer has demonstrated that the UPR signaling pathway is often activated by endocrine therapeutic interventions (60). Upon endoplasmic reticulum (EnR) stress, the chaperone protein GRP78 is released from sensor proteins and thus activates three distinct arms of the UPR pathway: IRE1-XBP1, PERK-eIF2α and ATF6. The pro-survival UPR pathway is temporarily activated to protect cells against EnR stress in normal cells; however, it is often constantly upregulated in cancer cells, promoting survival. The UPR components such as XBP1 and GRP78 are highly expressed in endocrine-resistant breast cancer cells that have undergone long-term exposure to tamoxifen or faslodex (61,62). Faslodex is a pure ER antagonist that activates the degradation of the receptor protein. The activated UPR is essential in regulating cell fate via modulation of apoptosis and autophagy in breast cancer cells (61). As the effects of long-term tamoxifen exposure on the induction of the UPR signaling pathway in breast cancer cells are often ER-independent, it is reasonable to predict that long-term tamoxifen exposure may induce UPR signaling in endometrial cells in a similar manner. The activated UPR signaling may promote cell survival and evasion of apoptosis, which are essential for cancer progression in endometrial tissue.

Consistent with this hypothesis, tamoxifen treatment induces the expression of EnR stress chaperone GRP78 in glandular and surface epithelia in human endometrial explants, indicating the activation of EnR stress/UPR signaling pathway in these cells (32). Our preliminary data, showing that the endometrial tissue of long-term tamoxifen-treated rats expresses higher levels of UPR component genes phosphor-eIF2α and CHOP compared with non-tamoxifen-treated control rats (unpublished data), also support this hypothesis. In breast cancer cells, GRP78 has been shown to modulate mTOR activity to promote autophagy (61). A number of studies have suggested that the mTOR signaling pathway is important in tamoxifen-associated endometrial cancer. The mean expression of mTOR in tamoxifen-associated endometrial cancer patients was significantly higher compared with the non-tamoxifen group (63). Addition of the mTOR inhibitor RAD001 (Everolimus) can prevent tamoxifen-associated endometrial hyperplasia in mice, and significantly decreases proliferating cell nuclear antigen staining promoted by tamoxifen in the epithelial and glandular cells (64). In addition, RAD001 decreases endometrial stromal cell proliferation in the tamoxifen-treated mice, which is known to send paracrine signals to promote the proliferation of endometrial epithelial cells (64). Further investigation to determine the role of EnR stress-UPR-mTOR-autophagy signaling pathway in tamoxifen-associated endometrial cancer, particularly following long-term exposure, is urgently required to provide insight for the development of cancer prevention strategies for patients taking tamoxifen.

The blockage of pathways that are essential for endocrine resistance in breast and tamoxifen-associated endometrial carcinogenesis is of tremendous value in clinical cancer prevention. As tamoxifen is a proven effective therapeutic and preventive drug for breast cancer, targeting the two major hurdles that limit its clinical usage is of significant benefit to breast cancer patients. The EnR stress-UPR signaling pathway may be an ideal target if proven to be critical for tamoxifen-associated endometrial cancer. In addition, accumulating evidence indicates that the physiology and homeostasis of the EnR and UPR signaling pathway are associated with obesity. Specifically, the IRE1α-XBP1 branch of the UPR pathway has been demonstrated to be crucial in glucose and lipid metabolism in addition to insulin function. Obesity is an established risk factor for both breast cancer and endometrial cancer. Therefore, in addition to the direct effects on endocrine resistance in breast cancer and tamoxifen-induced endometrial cancer, blocking the UPR pathway has the potential to benefit tamoxifen users indirectly through inhibition of obesity.

## 8. Conclusions

Tamoxifen is the most widely used breast cancer therapy and preventative drug worldwide. Understanding the molecular mechanisms of tamoxifen-promoted endometrial cancer is essential to identify strategies to lower the risk of developing endometrial cancer for breast cancer patients being treated with tamoxifen. Since tamoxifen metabolites may be genotoxic, tamoxifen functions primarily through non-genomic pathways to promote endometrial carcinogenesis. The modulation of estrogenic pathways is important in mediating the cell proliferation promoting effects of tamoxifen in endometrial cells. In addition, tamoxifen is known to regulate gene targets that are independent of estrogen. However, the tamoxifen-specific gene targets or networks that are essential for long-term tamoxifen exposure-induced endometrial cancer remain to be determined. Recent studies and our own data indicate that UPR and mTOR signaling may be significant in long-term tamoxifen exposure-induced endometrial cancer. As these two pathways are also major contributors to the mediation of endocrine resistance in breast cancer cells, targeting them may benefit tamoxifen-treated breast cancer patients by reducing endocrine resistance, as well as endometrial cancers following long-term usage. Larger scale clinical gene profile data and long-term tamoxifen experiments with *in vitro* and *in vivo* animal studies are needed to further clarify the involvement of these two pathways and molecular mechanisms that are essential for tamoxifen-associated endometrial cancer.
